# Association of Deferred vs Immediate Cord Clamping With Severe Neurological Injury and Survival in Extremely Low-Gestational-Age Neonates

**DOI:** 10.1001/jamanetworkopen.2019.1286

**Published:** 2019-03-29

**Authors:** Abhay Lodha, Prakesh S. Shah, Amuchou Singh Soraisham, Yacov Rabi, Ayman Abou Mehrem, Nalini Singhal

**Affiliations:** 1 Department of Community Health Sciences, University of Calgary, Calgary, Alberta, Canada; 2 Department of Pediatrics, University of Calgary, Calgary, Alberta, Canada; 3 Alberta Children’s Hospital Research Institute, University of Calgary, Calgary, Alberta, Canada; 4 Department of Pediatrics, University of Toronto, Toronto, Ontario, Canada; 5 Department of Pediatrics, Maternal-Infant Care Research Centre, Mount Sinai Hospital, Toronto, Ontario, Canada

## Abstract

**Importance:**

Deferred cord clamping (DCC) is recommended for term and preterm neonates to reduce neonatal complications. Information on the association of DCC with outcomes for extremely low-gestational-age neonates is limited.

**Objective:**

To compare neonatal outcomes after DCC and immediate cord clamping (ICC) in extremely low-gestational-age neonates.

**Design, Setting, and Participants:**

In this retrospective cohort study, eligible neonates born between January 1, 2011, and December 31, 2015, were divided into 2 groups: DCC and ICC. Neonates were recruited from tertiary neonatal intensive care units participating in the Canadian Neonatal Network, and analysis began in January 2018. Neonates were eligible if they were born at 22 to 28 weeks’ gestational age and admitted to a participating Canadian Neonatal Network neonatal intensive care unit during the study period. Neonates who were born outside a tertiary-level neonatal intensive care unit, were moribund at birth, needed palliative care before delivery, had major congenital anomalies, or lacked cord clamping information were excluded.

**Main Outcomes and Measures:**

Composite of severe neurological injury (intraventricular hemorrhage grade ≥3 with or without persistent periventricular echogenicity) or mortality before discharge.

**Results:**

Of 8221 admitted neonates, 4680 were included in the study, of whom 1852 (39.6%) received DCC and 2828 (60.4%) received ICC. There were 974 (52.7%) male neonates in the DCC group and 1540 (54.5%) male neonates in the ICC group. Median (interquartile range) gestational age was 27 (25-28) weeks for the DCC group and 26 (25-27) weeks for the ICC group. Median (interquartile range) birth weight was 930 (760-1120) g and 870 (700-1060) g for DCC and ICC groups, respectively. Neonates who received DCC had significantly reduced odds of the composite outcome of severe neurological injury or mortality (adjusted odds ratio [AOR], 0.80; 95% CI, 0.67-0.96), mortality (AOR, 0.74; 95% CI, 0.59-0.93), and severe neurological injury (AOR, 0.80; 95% CI, 0.64-0.99). The odds of bronchopulmonary dysplasia (AOR, 1.00; 95% CI, 0.84-1.19), retinopathy of prematurity stage 3 or higher (AOR, 0.94; 95% CI, 0.71-1.25), necrotizing enterocolitis stage 2 or higher (AOR, 0.86; 95% CI, 0.66-1.12), late-onset sepsis (AOR, 1.02; 95% CI, 0.85-1.22), and receipt of 2 or more blood transfusions (AOR, 0.93; 95% CI, 0.79-1.10) did not differ between the groups. Propensity score–matched analyses revealed lower odds of mortality (AOR, 0.79; 95% CI, 0.65-0.95), late-onset sepsis (AOR, 0.81; 95% CI, 0.69-0.95), and treatment for hypotension (AOR, 0.75; 95% CI, 0.60-0.95) in the DCC group.

**Conclusions and Relevance:**

In this study of extremely low-gestational-age neonates who received DCC or ICC, DCC was associated with reduced risk for the composite outcome of severe neurological injury or mortality.

## Key Points

**Question:**

Is deferred cord clamping associated with reduced odds of severe neurological injury or mortality in extremely low-gestational-age neonates?

**Findings:**

In this cohort study of 4680 extremely preterm neonates (22-28 weeks of gestation), 1852 received deferred cord clamping and 2828 received immediate cord clamping. Neonates who received deferred cord clamping had lower odds of severe neurological injury or mortality than neonates who received immediate cord clamping.

**Meaning:**

In extremely low-gestational-age neonates, deferred cord clamping is associated with reduced risk for severe neurological injury or mortality.

## Teaser

This cohort study compares neonatal outcomes after deferred cord clamping vs immediate cord clamping in extremely low-gestational-age neonates.

## Introduction

There is no physiological rationale for clamping the umbilical cord immediately after birth,^1^ although it was previously thought to be an important part of the active management of the third stage of labor.^2^ In moderate and late preterm infants, deferred cord clamping (DCC) reduces the need for transfusions, leads to circulatory stability, and improves blood pressure. Deferred cord clamping also decreases the odds of intraventricular hemorrhage, late-onset sepsis, and necrotizing enterocolitis (NEC).^3,4^ It has been suggested that DCC prevents low cardiac output state, increases right ventricular output and superior vena cava flow, protects vital organs, and avoids vigorous resuscitation, leading to the commencement of spontaneous breathing with better lung aeration and postnatal myocardial adaptation.^5,6,7^

A 2008 randomized clinical trial (RCT)^8^ and a 2004 meta-analysis^3^ have suggested some benefits of DCC in neonates; however, a large 2017 RCT^9^ that included infants born earlier than 30 weeks of gestation showed no difference in the combined outcome of death or major morbidity between the DCC and immediate cord clamping (ICC) groups. The authors acknowledged that their study was not powered to measure mortality, had a higher rate of nonadherence to DCC, and lacked information on maternal receipt of antenatal corticosteroids.^9^

A 2018 systematic review and meta-analysis^10^ of 18 DCC studies identified only 3^9,11,12^ that included neonates born at 28 weeks of gestation or earlier. The review demonstrated a reduced incidence of hospital mortality and blood transfusions after DCC. A 2016 systematic review and meta-analysis^13^ of 6 studies of neonates born earlier than 30 weeks of gestation (n = 299) reported that there was a lack of evidence to support DCC in this population. The American College of Obstetricians and Gynecologists^14^ recommends DCC for at least 30 to 60 seconds, and the International Liaison Committee on Resuscitation suggests DCC (>30 seconds) in preterm infants without specifying whether it has shown beneficial effects in extremely low-gestational-age neonates (ELGANs), in whom the need for initiation of resuscitation is paramount. At many centers, clinicians are still reluctant to adopt DCC for ELGANs in the absence of strong evidence because the ideal duration of DCC is unknown and they fear interfering with resuscitation.^15,16^

Deferred cord clamping has been variably adopted during resuscitation of ELGANs at some centers, although evidence of beneficial effects is lacking. The objective of our study was to determine the association of DCC with neonatal outcomes in ELGANs (22-28 weeks of gestation) admitted to the neonatal intensive care units (NICUs) of participating hospitals in the Canadian Neonatal Network (CNN). The purpose was to provide contemporary, real-life experience from an unrestricted national cohort.

## Methods

### Study Design and Study Population

This retrospective cohort study included ELGANs (22-28 weeks of gestation) admitted to CNN-affiliated NICUs between January 1, 2011, and December 31, 2015. The CNN collects data from all tertiary NICUs in Canada. Neonates were excluded if they met any of the following criteria: birth outside a tertiary-level NICU, moribund at birth, designated as needing palliative care before delivery, had major congenital anomalies, or lacked cord clamping information. This study is reported according to the Strengthening the Reporting of Observational Studies in Epidemiology (STROBE) reporting guideline.^17^ The University of Calgary research ethics board approved the study. Informed consent from parents was waived by the research ethics board. The primary data collection for the CNN was approved by the research ethics board of each hospital or quality improvement initiatives. The steering committee of the CNN approved the study protocol. Neonates who were born at 22 to 28 weeks of gestation and received DCC or ICC at birth were identified through the CNN database. The neonates were divided into 2 groups: DCC (cord clamping deferred in ≥30 seconds after birth) and ICC (cord clamped in ≤10 seconds sfter birth).

### Data Collection

We extracted information on maternal and neonatal demographic factors, including delivery room management information. Data analysis began January 2018. Information on mortality and other major morbidities, including severe neurological injury (SNI), retinopathy of prematurity (ROP), NEC, bronchopulmonary dysplasia (BPD), and late-onset sepsis, was collected using standardized forms and uploaded to the CNN database by each participating center. The CNN database has shown very good reliability in data abstraction.^18^

### Outcomes

The primary outcome was a composite outcome of SNI and mortality before discharge from the NICU. The secondary outcomes included mortality before discharge, SNI, BPD, ROP, NEC, late-onset sepsis, symptomatic hypotension, receipt of 2 or more blood transfusions, duration of mechanical ventilation, duration of continuous positive airway pressure, and length of stay. Severe neurological injury was defined as intraventricular hemorrhage grade 3 or higher and/or persistent periventricular echogenicity. Necrotizing enterocolitis was defined according to the modified Bell criteria, and NEC stage 2 or higher was classified as medical or surgical.^19^ Retinopathy of prematurity was defined according to the International Classification of Retinopathy of Prematurity.^20^ Bronchopulmonary dysplasia was defined as oxygen dependency at a postmenstrual age of 36 weeks or at the time of transfer to a level II unit.^21^ Late-onset sepsis was defined as isolation of pathogenic organisms from either blood or cerebrospinal fluid in a symptomatic neonate after age 3 days.

### Statistical Analysis

Maternal and neonatal characteristics and neonatal outcomes were compared between groups. Frequencies (percentage) or medians (interquartile range) were reported. Significance between groups was assessed using Pearson χ^2^ test for categorical variables or a Wilcoxon rank sum test for continuous variables. Univariate and multivariable logistic analyses were used for primary and secondary outcomes. We assessed the association of birth characteristics, resuscitation characteristics, and center effect with outcomes using 4 statistical models. In the first model, we adjusted for prenatal variables that occurred prior to DCC, including maternal receipt of antenatal steroids, cesarean delivery, and gestational age. In the second model, we adjusted for Apgar score at 5 minutes of less than 7, the need for extensive cardiopulmonary resuscitation (chest compression for >30 seconds with or without epinephrine), and the administration of mechanical ventilation, in addition to variables in the first model. In the third model, in addition to variables in model 2, we adjusted for the center. Hierarchical model 4 was clustered by site and adjusted for prenatal variables, including maternal receipt of antenatal steroids, cesarean delivery, and gestational age. Adjusted odds ratios (AORs) and 95% CIs were estimated. Owing to the baseline differences between the 2 groups, propensity score–matched analysis was conducted. Propensity score was estimated by using a multivariable logistic regression model with gestational age, sex, mode of delivery, and maternal antenatal steroid use. Matching was performed using the SAS macro match.sas and was based on a caliper width of 0.2-fold the SD of the logit-transformed propensity scores. Association of the outcome with DCC vs ICC groups in matched sample was examined with logistic regression analyses using generalized estimating equations with an unstructured correlation. Odds ratios and 95% CIs were reported. All analyses were conducted using SAS version 9.4 (SAS Institute). *P *values were 2-tailed, and significance was set at a *P* value less than .05.

## Results

**Figure 1.**
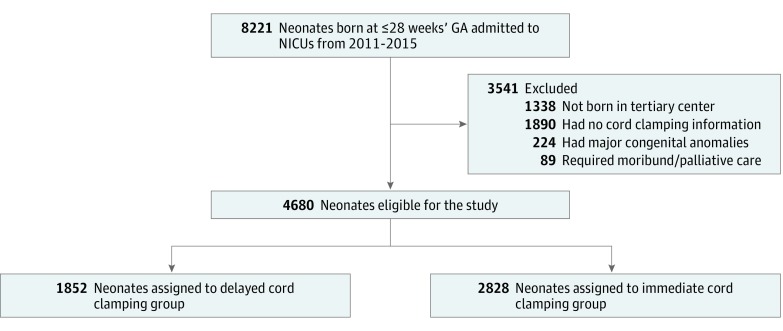
Flow Diagram of the Study Cohort GA indicates gestational age; NICU, neonatal intensive care unit.

**Figure 2.**
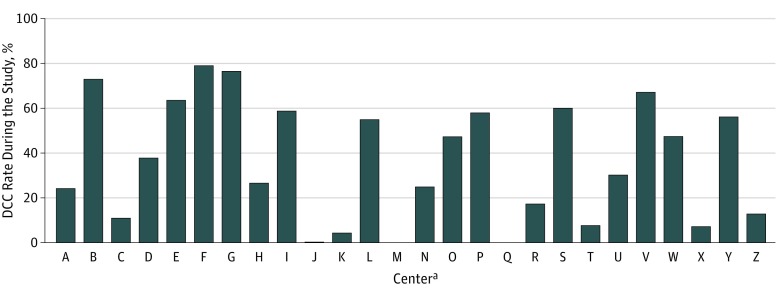
Center Variations in Deferred Cord Clamping (DCC) Rates in Canada ^a^Per policy, centers are represented by anonymized letters.

A total of 8221 ELGANs were admitted to the 26 participating NICUs between January 1, 2011, and December 31, 2015. A total of 3541 neonates were excluded from the study for various reasons (Figure 1). Of the 4680 eligible neonates, 1852 (39.6%) neonates had DCC and 2828 (60.4%) had ICC. The demographic characteristics of the neonates included in the study are shown in Table 1. There were 974 (52.7%) male neonates in the DCC group and 1540 (54.5%) male neonates in the ICC group. Median (interquartile range) gestational age was 27 (25-28) weeks for the DCC group and 26 (25-27) weeks for the ICC group. Median (interquartile range) birth weight was 930 (760-1120) g and 870 (700-1060) g for DCC and ICC groups, respectively. Deferred cord clamping rates varied from 0% to 79% among centers across Canada (Figure 2). Study population distribution in relation to DCC rates is shown in eTable 1 in the Supplement.

**Table 1.** Maternal and Neonatal Characteristics of Neonates Aged 22 to 28 Weeks of Gestation

Characteristic	No. (%)		
	DCC (n = 1852)	ICC (n = 2828)	*P* Value^a^
**Maternal Characteristics**			
Hypertension	292 (15.9)	439 (15.7)	.88
Antenatal steroid use	1768 (95.9)	2572 (91.9)	<.001
Cesarean delivery	984 (53.2)	1737 (61.5)	<.001
**Neonatal Characteristics**			
Male	974 (52.7)	1540 (54.5)	.24
GA, median (IQR), wk	27 (25-28)	26 (25-27)	<.001
GA group, wk			
<24	72 (3.9)	215 (7.6)	<.001
24	190 (10.3)	391 (13.8)	
25	271 (14.6)	475 (16.8)	
26	376 (20.3)	525 (18.6)	
27	410 (22.1)	590 (20.9)	
28	533 (28.8)	632 (22.4)	
Birth weight, median (IQR), g	930 (760-1120)	870 (700-1060)	<.001
Birth weight group, g			
≤1000	1113 (60.1)	1943 (68.7)	<.001
>1000	739 (39.9)	885 (31.3)	
Apgar score <7 at 5 min	670 (36.3)	1323 (46.9)	<.001
SNAP-II score, median (IQR)	14 (5-21)	14 (9-24)	<.001
Small for gestational age	152 (8.2)	272 (9.6)	.10
Need for ventilation by endotracheal tube at birth	805 (43.5)	1526 (54.0)	<.001
Extensive cardiopulmonary resuscitation (chest compression for >30 s, with or without epinephrine)	69 (3.7)	197 (7.0)	<.001
Respiratory distress syndrome	1538 (84.7)	2424 (88.7)	<.001
Surfactant administration	1249 (67.4)	2075 (73.4)	<.001
Patent ductus arteriosus	922 (50.5)	1664 (60.6)	<.001

Abbreviations: DCC, deferred cord clamping; GA, gestational age; ICC, immediate cord clamping; IQR, interquartile range; SNAP-II, Score for Neonatal Acute Physiology II.

^a^ Significance was assessed using a Wilcoxon rank sum test for continuous variables and Pearson χ^2^ test for categorical variables.

A higher proportion of mothers received antenatal steroids in the DCC group than in the ICC group (Table 1). The neonates in the DCC group were of later gestational age and had greater median birth weight than the neonates in the ICC group. The ICC group had a higher proportion of neonates born at 25 to 27 weeks of gestation than the DCC group. The neonates in the ICC group had significantly higher Score for Neonatal Acute Physiology II scores and were more likely to require ventilation with an endotracheal tube in the delivery room than the neonates in the DCC group. A higher proportion of neonates had a birth weight greater than 1000 g in the DCC group than the ICC group (Table 1). The children who were not included in the study owing to the absence of cord clamping information (n = 1890) are presented in eTable 2 in the Supplement. The data suggest that the neonates excluded from the analysis who were born in the tertiary centers and had no cord clamping information were of later gestational ages, received ventilator support at birth at higher rates, and received surfactant more frequently.

Table 2 shows a comparison of neonatal outcomes between the DCC and ICC groups. A smaller proportion of neonates in the DCC group received 2 or more blood transfusions than in the ICC group (535 [28.9%] vs 997 [35.3%], respectively). Fewer neonates in the DCC group were diagnosed as having patent ductus arteriosus, but there was no difference between the groups in the number of neonates discharged from tertiary centers on oxygen to level II centers. Table 2 also shows the association between DCC vs ICC groups after adjustment for confounders. Neonates who received DCC had significantly reduced odds of the composite outcome of SNI or mortality (AOR, 0.80; 95% CI, 0.67-0.96) and of mortality (AOR, 0.74; 95% CI, 0.59-0.93) and SNI (AOR, 0.80; 95% CI, 0.64-0.99) independently. There was no significant difference between the DCC and ICC groups with respect to BPD (AOR, 1.00; 95% CI, 0.84-1.19), ROP stage 3 or higher (AOR, 0.94; 95% CI, 0.71-1.25), NEC stage 2 or higher (AOR, 0.86; 95% CI, 0.66-1.12), late-onset sepsis (AOR, 1.02; 95% CI, 0.85-1.22), and receipt of 2 or more blood transfusions (AOR, 0.93; 95% CI, 0.79-1.10) (Table 2). Propensity score–matched analyses of 1783 matched pairs are reported in Table 2. These results were all favorable for the DCC group and were associated with significantly lower late-onset sepsis (AOR, 0.81; 95% CI, 0.69-0.95), treatment for hypotension (AOR, 0.75; 95% CI, 0.60-0.95), and mortality (AOR, 0.79; 95% CI, 0.65-0.95). Logistic regression with hierarchical model clustered by site also showed that neonates who received DCC had reduced odds of the composite outcome of SNI or mortality (AOR, 0.85; 95% CI, 0.73-0.98) and of mortality (AOR, 0.71; 95% CI, 0.55-0.92) and late-onset sepsis (AOR, 0.77; 95% CI, 0.62-0.96) individually (Table 2).

**Table 2.** Neonatal Outcomes: Univariate and Multivariable Analyses

Outcome	No. (%)		Adjusted OR (95% CI)				Propensity Score–Matched OR (95% CI)^e^
	DCC (n = 1852)	ICC (n = 2828)	Model 1^a^	Model 2^b^	Model 3^c^	Model 4^d^	
Composite outcome of severe neurological injury or mortality^f^	379 (20.5)	726 (25.7)	0.85 (0.73-0.98)^g^	0.91 (0.78-1.06)	0.80 (0.67-0.96)^g^	0.85 (0.73-0.98)^g^	0.89 (0.77-1.04)
Mortality	206 (11.1)	478 (16.9)	0.71 (0.59-0.86)^g^	0.77 (0.64-0.93)^g^	0.74 (0.59-0.93)^g^	0.71 (0.55-0.92)^g^	0.79 (0.65-0.95)^g^
Severe neurological injury^f^	230 (12.4)	396 (14)	0.96 (0.80-1.14)	1.01 (0.84-1.21)	0.80 (0.64-0.99)^g^	0.96 (0.77-1.19)	1.01 (0.83-1.22)
Bronchopulmonary dysplasia	540 (32.6)	851 (35.9)	0.93 (0.81-1.07)	0.99 (0.86-1.14)	1.00 (0.84-1.19)	0.93 (0.70-1.23)	0.99 (0.86-1.14)
Retinopathy of prematurity stage ≥3	148 (11.8)	245 (12.5)	1.07 (0.85-1.35)	1.11 (0.88-1.40)	0.94 (0.71-1.25)	1.07 (0.71-1.62)	1.27 (0.99-1.62)
Necrotizing enterocolitis stage ≥2	145 (7.8)	267 (9.5)	0.84 (0.68-1.05)	0.84 (0.68-1.04)	0.86 (0.66-1.12)	0.84 (0.66-1.08)	0.90 (0.71-1.14)
Late-onset sepsis	374 (20.2)	715 (25.3)	0.77 (0.67-0.89)^g^	0.78 (0.68-0.91)^g^	1.02 (0.85-1.22)	0.77 (0.62-0.96)^g^	0.81 (0.69-0.95)^g^
Jaundice treated with phototherapy	268 (14.5)	280 (10.0)	1.54 (1.29-1.85)^g^	1.58 (1.31-1.90)^g^	NA	1.54 (0.55-4.35)	1.58 (1.28-1.95)^g^
Hypotension treated with inotropes and fluid boluses	134 (7.2)	302 (10.7)	0.73 (0.59-0.91)^g^	0.81 (0.65-1.01)	NA	0.73 (0.50-1.07)	0.75 (0.60-0.95)^g^
Receipt of ≥2 blood transfusions	535 (28.9)	997 (35.3)	0.84 (0.74-0.96)^g^	0.88 (0.77-1.01)	0.93 (0.79-1.10)	0.84 (0.67-1.06)	0.90 (0.79-1.04)

Abbreviations: DCC, deferred cord clamping; ICC, immediate cord clamping; NA, not applicable; OR, odds ratio.

^a^ Model 1 was adjusted for prenatal variables, including maternal receipt of antenatal steroids, cesarean delivery, and gestational age.

^b^ Model 2 was adjusted for prenatal and immediate postnatal variables: model 1 variables, Apgar score at 5 minutes lower than 7, need for extensive cardiopulmonary resuscitation, and need for mechanical ventilation.

^c^ Model 3 was adjusted for model 2 variables and center.

^d^ Hierarchical model 4 clustered by site was adjusted for maternal receipt of antenatal steroids, cesarean delivery, and gestational age.

^e^ Propensity score–based adjusted for gestational age, sex, mode of delivery (cesarean delivery vs vaginal delivery), and maternal receipt of antenatal steroids. There were 1783 pairs in each group included in the analysis.

^f^ Severe neurological injury includes intraventricular hemorrhage grade 3 and greater with or without persistent periventricular echogenicity.

^g^ *P* < .05.

After the identification of differences in mortality, we investigated the causes of mortality between the 2 groups using post hoc analyses. Table 3 shows the most frequent causes of mortality in the 2 groups. The data about cause of mortality were taken from death certificates and extracted by abstractors. There was a significantly higher incidence of mortality due to SNI in the ICC group than in the DCC group (93 [19.5%] vs 22 [10.7%]; risk difference, 9%; 95% CI, 3%-14%; *P* = .005).

**Table 3.** Causes of Mortality

Cause of Mortality	No. (%)		*P* Value
	DCC (n = 206)	ICC (n = 478)	
Respiratory	64 (31.1)	121 (25.3)	.12
Severe RDS	14 (6.8)	12 (2.5)	.007
PPHN	15 (7.3)	23 (4.8)	.20
Pulmonary hemorrhage	13 (6.3)	27 (5.7)	.74
Pulmonary hypoplasia	9 (4.4)	20 (4.2)	.91
Severe BPD	7 (3.4)	25 (5.2)	.30
Other	6 (2.9)	14 (2.9)	.99
Late-onset sepsis	42 (20.4)	76 (15.9)	.15
Gastrointestinal disorders	36 (17.5)	83 (17.4)	.97
NEC stage ≥2	31 (15.1)	77 (16.1)	.73
Spontaneous intestinal perforation	4 (1.9)	5 (1.1)	.46
Other	1 (0.5)	1 (0.2)	.51
Severe neurological injury^a^	22 (10.7)	93 (19.5)	.005
Extreme prematurity	21 (10.2)	56 (11.7)	.56
Refractory hypotension	5 (2.4)	8 (1.7)	.55
Multiorgan failure	6 (2.9)	9 (1.9)	.40
Other	10 (4.9)	32 (6.7)	.36

Abbreviations: BPD, bronchopulmonary dysplasia; DCC, deferred cord clamping; ICC, immediate cord clamping; NEC, necrotizing enterocolitis; PPHN, persistent pulmonary hypertension of the newborn; RDS, respiratory distress syndrome.

^a^ Severe neurological injury includes intraventricular hemorrhage grade 3 and greater with or without persistent periventricular echogenicity.

## Discussion

In this large, population-based cohort study of ELGANs, we found that DCC was associated with a reduction in the composite outcome of SNI or mortality and the individual outcomes of mortality and SNI. However, after adjusting for potential confounders, there was no difference between the DCC and ICC groups in the odds of other morbidities, including BPD, ROP stage 3 or higher, NEC stage 2 or higher, late-onset sepsis, and receipt of 2 or more blood transfusions. The propensity score–based analyses did not show a statistically significant difference in the composite outcome of SNI or mortality. However, outcomes were favorable for the DCC group and revealed lower odds of mortality, late-onset sepsis, and treatment for hypotension in the DCC group. Among the neonates who died, SNI was more likely to be the most common cause of death in the ICC group. In the DCC cohort, a higher proportion of mothers received antenatal steroids, neonates were of later gestational age and had greater median birth weight, a higher proportion of neonates were born after 25 weeks of gestation, and the neonates had lower Score for Neonatal Acute Physiology II scores and were less likely to require ventilation with an endotracheal tube in the delivery room than the neonates in the ICC group. Overall, preterm neonates in the DCC group were healthier compared with neonates in the ICC group. This may introduce residual confounding, and to address it to some extent, we conducted analyses using several different techniques. We speculate that our findings could be owing to improved cardiovascular stability and reduced fluctuations in cerebral blood flow, a lower number of fluid boluses, increased superior vena cava blood flow, and stable blood pressure after DCC.^22,23,24,25,26,27^

We identified reduced odds of mortality in our study after DCC. This finding is in contrast with results from a 2017 RCT (relative risk, 0.69; 95% CI, 0.49-0.97; unadjusted *P* = .03; adjusted *P* = .39 after post hoc adjustment for multiple secondary comparisons)^9^ and consistent with results from a 2018 meta-analysis (relative risk, 0.68; 95% CI, 0.52-0.90).^10^ Our study confirms these findings in a pragmatic cohort of 4680 neonates. Additionally, we performed a detailed evaluation of the causes of mortality and found that, with the exception of SNI, there were no differences in the most frequent causes of mortality between the DCC and ICC groups. We believe that SNI per se does not result in immediate mortality unless there is massive exsanguination; however, it may lead to decisions to opt for comfort care. Although this finding corresponds with our overall finding of reduced SNI in the DCC group, our findings should be further corroborated in future studies. We believe that DCC could be a strategy for improving the overall survival of ELGANs. Some of the limitations addressed in our study include detailed and complete information on maternal receipt of antenatal steroids and use of a pragmatic cohort based on what was actually received, rather than an intention-to-treat approach typical of an RCT and unselected cohorts. Our study has complete information about maternal receipt of antenatal corticosteroids and the primary outcome of mortality or SNI based on neonates included in the DCC vs ICC groups, compared with a 2017 RCT^9^ in which infants were not adhered to the DCC group and primary outcome was based on mortality or any multiple morbidities.

The baseline characteristics between groups in our study identified some important differences. We generated 4 models to determine the association of several prenatal and postnatal predictors with the outcome estimates. The results of these models were mostly similar but differed somewhat among the 4 models. Models 1 and 2 identified some interesting associations of DCC with late-onset sepsis, hypotension requiring inotropes, and receipt of 2 or more blood transfusions, which have also been noted in previous studies.^11,26,27,28,29,30^ These findings need to be evaluated in future studies. In the third model, which was adjusted according to medical center as well as prenatal and immediate postnatal characteristics, we did not identify an association of DCC with BPD, severe ROP, NEC, late-onset sepsis, or receipt of 2 or more blood transfusions. The final hierarchical model clustered by site also showed that neonates who received DCC had reduced odds of the composite outcome of SNI or mortality as well as mortality and late-onset sepsis individually.

Additionally, we observed that neonates in the DCC group required more phototherapy than neonates in the ICC group (model 2), which was likely because the extra volume of blood in neonates in the DCC group resulted in overproduction of bilirubin. This finding is consistent with previous reports that identified higher peak bilirubin levels in DCC groups.^8,10,31,32^ However, the implications of these findings are unclear, as the incidence of high peak bilirubin levels has not convincingly translated into an increased need for phototherapy or exchange transfusions.^29,33,34,35^

After adjusting for multiple factors in the models, the odds of BPD, ROP, and NEC were not significantly reduced in the DCC group, which is consistent with other studies.^9,15,27,30,36,37^ Our finding that DCC was associated with reduced odds of late-onset sepsis in some models and not in others is similar to that reported by Mercer et al.^27^ It is possible that there was a placental transfusion of a higher concentration of hematopoietic progenitor cells in preterm infants that may have provided extra immunological competence.^27,38^ However, this finding should be further evaluated.

### Strengths and Limitations

Our study has several strengths. This is one of the largest population-based, pragmatic cohorts in preterm infants born at or before 28 weeks of gestation, to our knowledge. The use of uniform variable definitions across various CNN sites limited potential errors and variability. The results of our study are based on a national cohort and are therefore generalizable. We were able to overcome limitations of RCTs, such as adjusting for maternal receipt of antenatal steroids, including neonates who actually received ICC or DCC, and using a large sample to evaluate outcomes.

The study had the following limitations. First, several neonates were excluded from the study owing to a lack of information on the timing of cord clamping. There were differences in certain characteristics of these neonates; most notably, they had later gestational ages. Second, we do not have information on the potential reasons for performing ICC. We believe that there is a potential risk of selection bias in the neonates included in the ICC group because some of these neonates were suspected to need extensive support at birth. It is possible that the neonates who received DCC were planned births (as supported by the higher rates of maternal antenatal steroid use) and may have had relatively stable intrapartum periods. Third, this was a retrospective analysis; thus, the study has inherent biases and residual confounding factors. Fourth, we also noticed that there was marked center variability in the rates of DCC, indicating slower uptake, skepticism of benefit, lack of personnel providing support during the waiting period, or following of traditional protocols. Fifth, we did not have information on exact durations of DCC, which has been identified as a deficiency of the study. We have since started collecting this information in our database (beginning in January 2016).

## Conclusions

In conclusion, we identified that in ELGANs, DCC was associated with a reduction in the composite outcome of SNI or mortality. There were also reduced odds of mortality and SNI individually in some models. The association of DCC with late-onset sepsis and fluid administration may be favorable; however, further studies are needed. The findings of recent RCTs and meta-analyses, as well as our findings, need to be considered in the determination of future directions, as they may have implications for clinical practice. Further research should be conducted to answer questions associated with optimal timing, optimal positioning to allow placental transfusion, interim measures (eg, provision of resuscitation while waiting for cord clamping), and the impact of placental transfusion on parental wishes and volume for cord blood banking.

## Notes

Supplement. **eTable 1.** Population and Deferred Cord Clamping Rates by Site **eTable 2.** Maternal and Neonatal Characteristics of Neonates Excluded Because of Absence of Cord Clamping Information
