# Supplementary material for: Association of Deferred vs Immediate Cord Clamping With Severe Neurological Injury and Survival in Extremely Low-Gestational-Age Neonates
Source: JAMA Netw Open. 2019 Mar 29;2(3):e191286. doi: 10.1001/jamanetworkopen.2019.1286 (PMC6450317; doi:10.1001/jamanetworkopen.2019.1286)
Supplement: Supplement. — eTable 1. Population and Deferred Cord Clamping Rates by Site eTable 2. Maternal and Neonatal Characteristics of Neonates Excluded Because of Absence of Cord Clamping Information [file jamanetwopen-2-e191286-s001.pdf]

Supplementary Online Content

Lodha A, Shah PS, Soraisham AS, Rabi Y, Abou Mehren A, Singhal N; Canadian Neonatal Network Investigators. Association of deferred vs immediate cord clamping with severe neurological injury and survival in extremely low-gestational-age neonates. *JAMA Netw Open*. 2019;2(3):e191286. doi:10/1001/jamanetworkopen.2019.1286

eTable 1. Population and Deferred Cord Clamping Rates by Site

eTable 2. Maternal and Neonatal Characteristics of Neonates Excluded Because of Absence of Cord Clamping Information

This supplementary material has been provided by the authors to give readers additional information about their work.

© 2019 Lodha A et al. *JAMA Network Open.*

eTable 1. Population and Deferred Cord Clamping Rates by Site

Site*	Number of eligible preterm neonates	Deferred cord clamping rate (%)
**A**	255	24.31
**B**	231	73.16
**C**	91	10.99
**D**	29	37.93
**E**	518	63.71
**F**	143	79.02
**G**	81	76.54
**H**	323	26.63
**I**	131	58.78
**J**	341	0.29
**K**	111	4.5
**L**	80	55
**M**	263	0
**N**	16	25
**O**	639	47.4
**P**	295	58.06
**Q**	139	0
**R**	89	17.27
**S**	78	59.55
**T**	43	7.69
**U**	112	30.23
**V**	37	66.96
**W**	154	47.4
**X**	14	7.14
**Y**	432	56.25
**Z**	31	12.9

*Per Canadian Neonatal Network policy, site names are represented by anonymized letters.

© 2019 Lodha A et al. *JAMA Network Open.*

eTable 2. Maternal and Neonatal Characteristics of Neonates Excluded Because of Absence of Cord Clamping Information

Characteristics	Included neonates (n=4680)	Excluded neonates[a] (n=1890)	*P* value[b]
**Maternal Characteristics**			
Maternal hypertension, n (%)	731 (15.8)	337 (18.2)	.018
Antenatal steroid, n (%)	4340 (93.5)	1692 (91.6)	.007
Cesarean section, n (%)	2721 (58.2)	1122 (59.8)	.239
**Neonatal Characteristics**			
GA, median (IQR)	26 (25, 27)	26 (25, 28)	.004
GA group, n (%)			
23	287 (6.1)	104 (5.5)	.082
24	581 (12.4)	207 (10.9)	
25	746 (15.9)	293 (15.5)	
26	901 (19.3)	349 (18.5)	
27	1000 (21.4)	403 (21.3)	
28	1165 (24.9)	534 (28.3)	
Birth weight, median (IQR)	890 (720, 1090)	890 (720, 1090)	.863
Birth weight group, n (%)			
≤1000 grams	3056 (65.3)	1236 (65.4)	0.940
>1000 grams	1624 (34.7)	654 (34.6)	
Apgar score at 5 min <7, n (%)	1993 (42.7)	789 (42.1)	.637
SNAP-II score, median (IQR)	14 (9, 22)	14 (9, 24)	.162
Male, n (%)	2514 (53.8)	996 (52.9)	.504
Small for gestational age, n (%)	424 (9.1)	189 (10.0)	.226
Need for ventilation by endotracheal tube at birth, n (%)	2331 (49.8)	1156 (61.8)	<.001
Extensive cardiopulmonary resuscitation (chest compression for >30 seconds ± epinephrine), n (%)	266 (5.7)	119 (6.4)	.292
Respiratory distress syndrome, n (%)	3962 (87.1)	1554 (86.4)	.478
Surfactant administration, n (%)	3324 (71.0)	1417 (75.0)	<.001
Patent ductus arteriosus, n (%)	2586 (56.6)	1014 (55.7)	.553

[a]Group includes neonates excluded because of absence of cord clamping information or because they were not born in tertiary centers only. Does not include neonates excluded because they were moribund, on palliative care, or had major congenital anomalies.

[b]Significance was determined using Wilcoxon rank tests for continuous variables and Pearson's chi-square tests for categorical variables.

Abbreviations: GA, gestational age; IQR, interquartile range; SNAP-II, score for neonatal acute physiology-II

© 2019 Lodha A et al. *JAMA Network Open.*
